# A qualitative investigation of stressful life events and mental health disorders: The views of patients and caregivers in Bangladesh

**DOI:** 10.1371/journal.pone.0281649

**Published:** 2023-02-09

**Authors:** Nishad Nasrin, Tunvir Ahamed Shohel, Taufiq-E-Ahmed Shovo, Fariha Farjana, Hamalna Nizam, Morsheda Akter Heme, Ashraful Islam, Pranto Paul, Md. Tanvir Hossain

**Affiliations:** 1 Economics Discipline, Social Science School, Khulna University, Khulna, Bangladesh; 2 Sociology Discipline, Social Science School, Khulna University, Khulna, Bangladesh; 3 English Discipline, Arts and Humanities School, Khulna University, Khulna, Bangladesh; Universidad Internacional de La Rioja, SPAIN

## Abstract

**Background:**

Mounting mental health disorders among students has become a significant challenge in producing quality graduates with bright minds. Therefore, it is crucial to investigate the underlying causes of students’ mental health-related problems and their experiences while living with mental health disorders. This study investigates the causes and exposures of mental health problems among university students in Bangladesh. For this purpose, a qualitative method was used, and an unstructured in-depth interview schedule was used to collect data from students and caregivers. The students were selected from Khulna University, and data were collected in two consecutive months, i.e., November and December 2021. Using a convenient sampling technique, eight (8) students with mental health issues and five (5) caregivers were interviewed to obtain the necessary data.

**Results:**

The findings showed that the intertwined social circumstances led to mental health problems among university students. The students experienced deep depression following a breakup of their romantic affairs/relationships. The prolonged isolation or social distancing due to the government-imposed strict lockdown during the COVID-19 also produced frustration regarding the possible loss of both academic and professional careers. Furthermore, the growing academic pressure in a form of an unfamiliar approach to teaching and learning—online education–also compelled students to complain about depression and suicidal tendencies as parts of their experiences.

**Conclusions:**

This study recommends that the government and policymakers prioritize mental health issues in educational institutions, and they should enforce specific strategies, such as introducing age-specific mental health services and student counseling at educational institutions to reduce growing mental health issues. Furthermore, a positive approach from the family and community is also required to battle against mental health disorders. Besides, nationally representative empirical research is recommended to comprehend the growing mental health issues among students in the 21^st^ century to figure out solutions for the present and the future.

## Background

Mental health disorders among young adults have become a major concern for psychiatrists in Bangladesh. The most recent national mental health survey in Bangladesh suggested that around 15% of young adults are suffering from different mental health disorders, and the most common problems are depression and anxiety [1]. The situation deteriorated further during the COVID-19 pandemic. Studies in Bangladesh indicated that young adults, irrespective of sex and residence, have been experiencing heightened depression, anxiety, stress, sleeping disorders, and fear of COVID-19 during the pandemic [2–6]. Prolonged home confinement, exposure to misinformation on social and electronic media, uncertainty over academic and professional life, financial insecurity, and unemployment triggered growing mental health disorders among young adults in Bangladesh during the pandemic [3,6–9]. A similar situation is observed in other countries in the Indian sub-continent and elsewhere. For example, in Pakistan, studies suggested an intensified prevalence of mental health issues, including anxiety and depression symptoms, among students [10,11] as well as other age groups [12] during the COVID-19 pandemic.

In addition to the unprecedented COVID-19 pandemic, life events may also lead to mental health disorders among young adults. A Canadian study, for example, pointed out that stressful life events, including a romantic breakup, family disruption, troubled interpersonal communication, and personal stress, such as health and school-related issues, significantly influenced the mental health of young adults [13]. A study on African-American adolescent girls receiving psychiatric care showed that heterosexual romantic dating experience and peer relationships were the strongest determinants of disruptive behavior and self-silencing [14]. Another North American study showed that child abuse and intimate partner violence significantly influenced the mental detachment of pregnant women from their partners, eventually leading to heightened anxiety and avoidance [15].

Apart from personal and family issues, a few studies stressed the relationship between academic achievement and mental stability. For example, a survey of university students in the UK indicated that loneliness, academic stressors, psychosocial issues, and a sense of coherence were the most potent predictors of mental wellbeing [16]. Another study in the USA found that academic stress led to the poor mental wellbeing of college students, particularly white and South Asian students, and the situation worsened further during COVID-19 [17]. In addition to the academic workload through emergency online learning, with growing academic uncertainty, increased sedentary behavior together with household responsibilities, particularly during financial hardship, may lead to potentially stressful life experiences and may increase the susceptibility to growing depression and anxiety among young adults [6,18–20], including early married adolescents [21].

Although the reasons leading to mental health issues among young adults during the pandemic are relatively well documented in Bangladesh [2–9] and elsewhere [10–12,19,20,22]; to the best of our knowledge, there is a shortage of empirical studies tracing back the reasons for mental health problems in general, particularly the individualized issues that are causing psychological problems and disrupting their lives [19]. Studies conducted in other countries have identified some predictors that may have negatively impacted young individuals’ mental conditions [13–18]. Hence, the current study aims to explore the underlying causes of mental disorders among young adults in Bangladesh in order to advocate timely solutions for young adults experiencing mental health problems to assure uninterrupted educational progress and to minimize negative academic and psychosocial outcomes through institutional interventions [19,20].

## Methods

### Research design and participants

Based on the objectives of the study, a qualitative approach was adopted to collect data from both the university students experiencing mental health problems as well as the caregivers who look after the patients during treatment (see Table 1). The qualitative method was used as it helps the researchers to describe human behavior, thinking, and action in a logical manner [23].

**Table 1:** Background information of the participants.

Serial No	Status	Sex	Age	Level of Education	Occupation	Income (BDT)	Causes
Participant 1	Patient	Male	22	Third Year, BSS (Honors)	Student & Private tutor	60,000	Breakup of relationship
Participant 2	Patient	Male	22	Fourth Year, BA (Honors)	Student & Private tutor	6,000	Academic pressure
Participant 3	Patient	Female	24	Second Year, Degree	Student & Tailor	2,000	Academic pressure
Participant 4	Patient	Female	24	Fourth Year, BSS (Honors)	Student & Housewife	No income	COVID-19 and lockdown situation
Participant 5	Patient	Male	24	Third Year, BSS (Honors)	Student	No income	Academic pressure
Participant 6	Patient	Male	23	Third Year, BSS (Honors)	Student	No income	COVID-19 and lockdown situation
Participant 7	Patient	Male	25	Third Year, BSS (Honors)	Student	No income	Breakup of relationship
Participant 8	Patient	Male	21	Second Year, BA (Honors)	Student	No income	Academic pressure
Participant 9	Caregiver	Female	43	SSC	Housewife	No income	
Participant 10	Caregiver	Female	48	Class 5	Housewife	No income	
Participant 11	Caregiver	Male	32	Degree	Businessman	15,000	
Participant 12	Caregiver	Male	48	BCom	Business	25,000	
Participant 13	Caregiver	Female	40	Degree	Housewife	No income	

### Ethics issues

This study was approved by the Ethical Clearance Committee of Khulna University, Bangladesh (Reference No. KUECC– 2021/05/31). An informed consent letter was attached in the interview schedule where the participants were provided with information concerning the research purpose, confidentiality, anonymity, and the right to revoke participation without prior justification. An informed consent was obtained from all subjects and/or their legal guardian(s). There was no incentive for the participants. Besides, all methods were carried out in accordance with relevant guidelines and regulations, and pseudonyms were used to anonymize the participants.

### Interview outline

After evaluating the relevant literature, a semi-structured interview schedule was developed that contained four mutually exclusive modules, where first two modules were focused on socio-demographic and household information to collect primary data from the research participants (see S1 Appendix). In the third module, student participants receiving mental healthcare were questioned about their mental health related histories as well as social response from their families and communities. In the final module of the interview schedule, the caretakers of students receiving mental healthcare were asked about their perspective of mental health experience, and how they dealt with it in a social setting where individuals with mental health problems are generally labelled by society [24].

### Sampling and data collection

**Fig 1:**
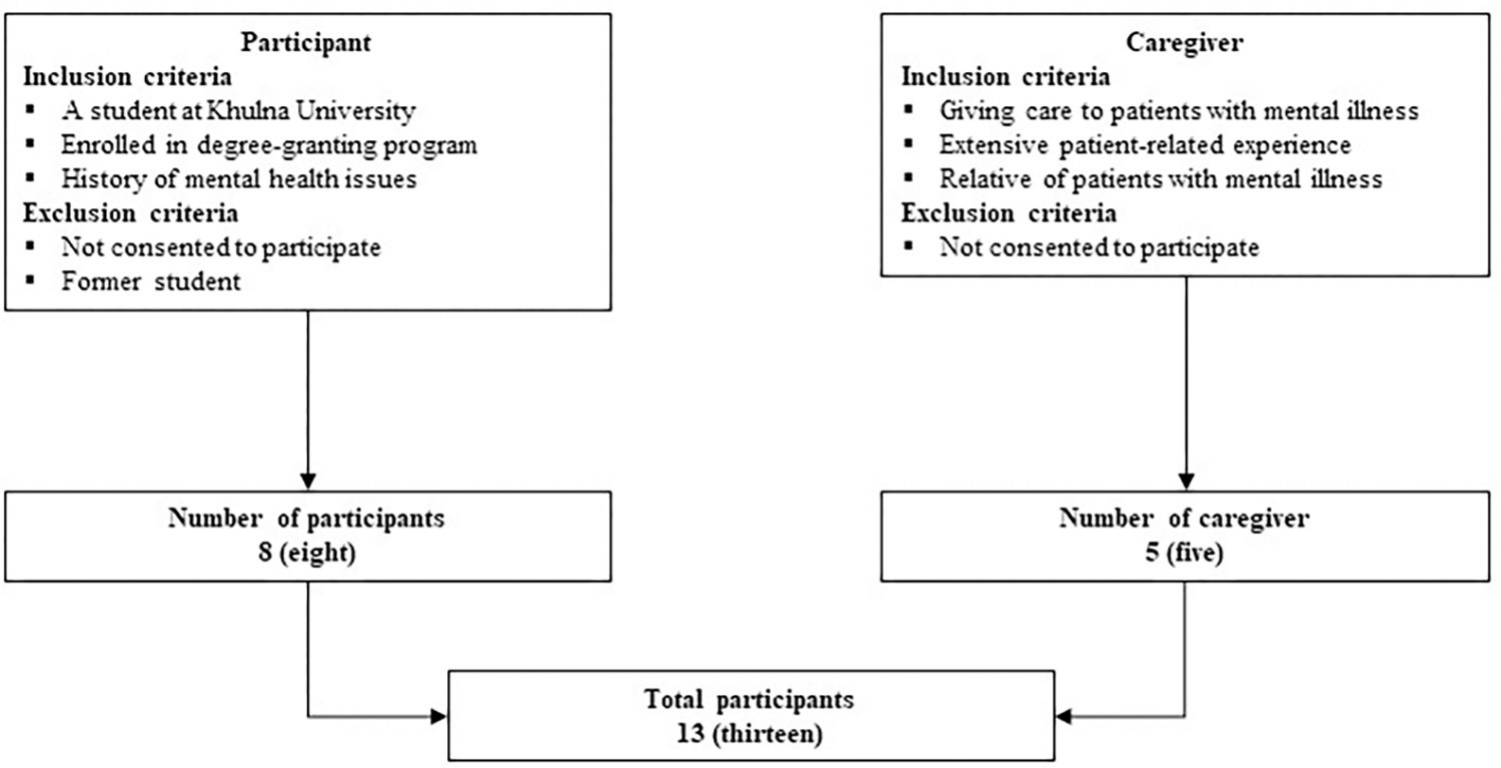
Selection of study participants.

There were two phases of the data gathering process in the current investigation, where face-to-face in-depth interviews (IDIs) were conducted with the research participants. However, the participants were selected based on some specifications; for example, student receiving mental healthcare were recruited considering three criteria: e.g., (i) they must be students, (ii) enrolled in a regular academic program, and (iii) must be receiving mental healthcare from psychiatrist(s); whereas for caregivers, there were two criteria: (i) must be a relative to students receiving mental healthcare, and (ii) must be taking care of the students with mental health issues and have a detailed knowledge about the ailment of the care-receiver. Considering the aforesaid specifications, the researchers approached the psychiatrists working in Khulna, and with the verbal consent from the students receiving mental healthcare and their guardians, the researchers approached the participants. Initially, eight (8) individuals with mental health issues were questioned over the course of two months November and December 2021. Next, five (5) caregivers who lived with and took care of the individuals with mental health issues were interviewed in detail in the latter part of the data collection phase (Fig 1). It is worth mentioning that due to the sensitive nature of the research issue, the sample size was very limited in number in order to assure confidentiality and anonymity of the students receiving mental healthcare and their caregivers. Furthermore, in qualitative research, it is possible to gain insight from minimal number of sample cases [25,26] and to make generalizations [27]. The interviews were done entirely in Bangla and were uninterrupted at any point. On average, each interview lasted between 40 to 50 minutes. Each interview was audio recorded in its entirety with the participants’ permission to do so. However, all interviews were kept confidential, and participants could stop participating in the study at any moment without being asked the reason.

### Reflexivity

The vulnerable situation of the research participants was acknowledged and recognized, hence before starting the interview, the participants were asked for their willingness to participate in the research work. Additionally, the interviewers were also aware of questions that might create unrest in the participants. To avoid such complexities, the interviewers tried to maintain a friendly relationship with the participants. Apart from this the interviewers kept a neutral position and followed the golden rule of unconditional acceptance principle to receive bias-free authentic information regarding the issue of investigation. Therefore, the interviewers have faith in the honesty and openness of the participants since the former have not seen any evidence to suggest otherwise.

### Data analysis

Using the qualitative data analysis software NVivo 12, the researchers classified and analyzed the interview transcripts into themes after completing the interviews. Each author was responsible for summing up key elements and creating the tone of the topics. Narrative and theme analyses were used to compile the results. By comparing how often each topic appeared, the researchers were able to draw a link between the study’s results and the real world. The researchers also discussed and agreed upon the interview data to include in the preliminary report after each round of interviews. Meanwhile, the interview data was thoroughly analyzed and debated by the study team in order to iron out any discrepancies and, in the worst-case scenario, rule them out entirely.

## Results

### The reason behind mental health issues (perspective of patients)

**Fig 2:**
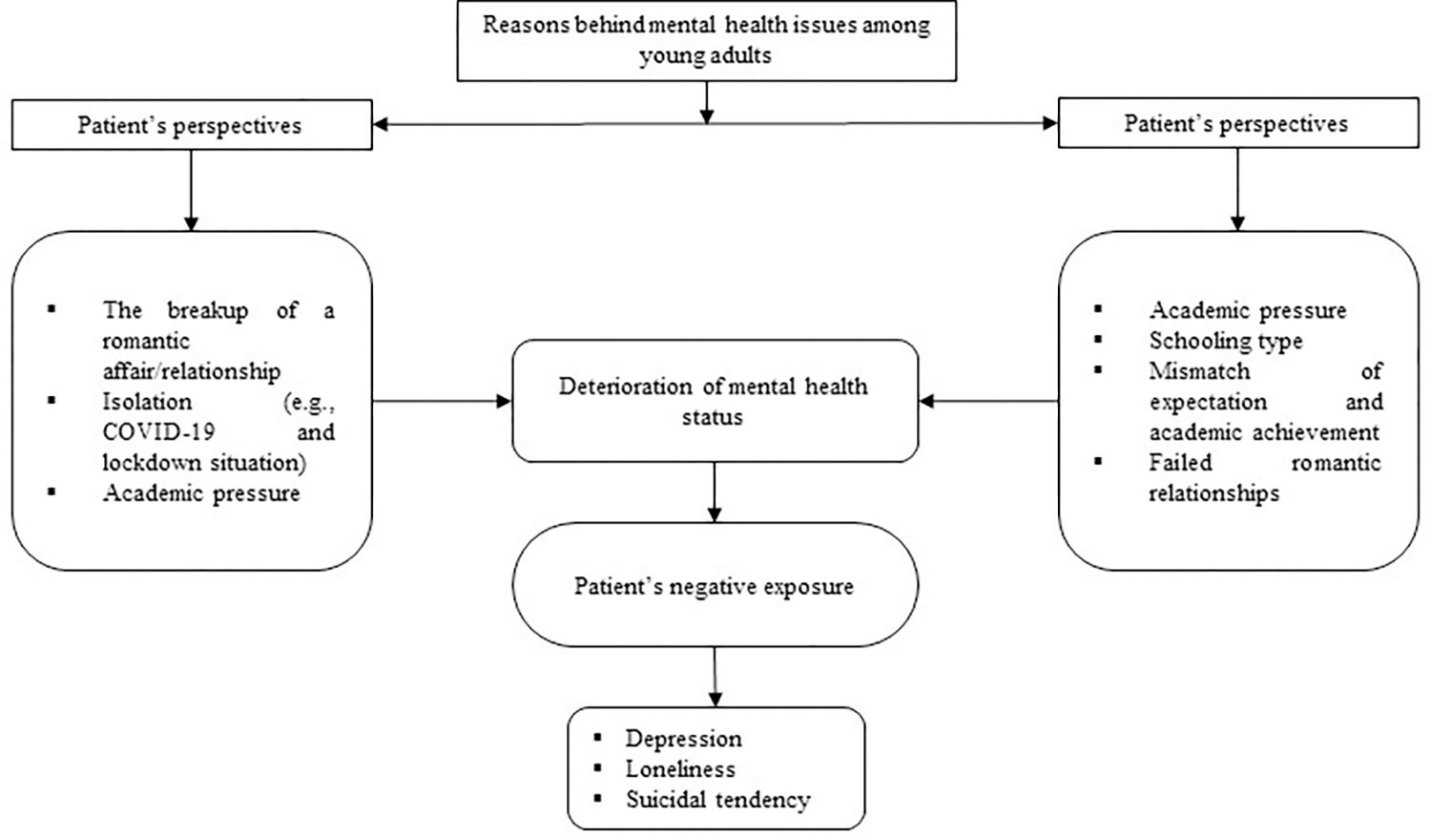
Thematic analysis of mental health issues among young adults.

The qualitative findings were composed of 13 in-depth interviews, and among them eight informants were directly mental health patients and the rest five were their closest caregivers. Based on our in-depth interview data and the experiences shared by our interviewees (see Fig 2), the reason behind patient’s mental health issues could be explained under three major themes:

1. The breakup of a romantic affair/relationship
2. COVID-19 and lockdown situation
3. Academic pressure

Breakup of relationship.

Among the informants with mental health problems, at least two of them reported that their mental health problems started after breaking up their affair or romantic relationship. Those who faced mental health problems in aftermath of the broken relationship went down to deep depression and frustration. Participant 1 (a 22-year-old university going student), shared his feelings in this way:

*“My girlfriend broke our relationship all of a sudden and I was not prepared to accept it. It felt like my heart was broken, and I found myself frustrated and under deep depression as an aftermath of the breakup.”*

Similarly, Participant 7 (a 25-year-old university going student), also explained his experiences in this way:

*“I realized my mental health problem started because of my breakup with my girlfriend. As we got separated, I found myself lonely and depressed afterwards.”*

COVID-19 and lockdown situation.

About our second theme of reason behind the mental health problems, our findings showed that global pandemic and lockdown situations are powerful enough to inflict individuals to mental health problems. Two of the mental health patients reported that their problems started with the spread of the COVID-19 pandemic all over Bangladesh and the lockdown situation imposed by the government. Sufferings such as interruption in formal education and study session, and loss of income created major mental health problems to the concerned individuals. In this regard, Participant 4 (a 24-year-old university going student), shared her experiences as followed:

*“My mental health condition got deteriorated with the introduction of the COVID-19 pandemic in Bangladesh and all the sufferings it introduces to our life. Regarding my study and its completion, I was depressed. As I see due to the COVID-19 pandemic, a lockdown was imposed, and all the educational institutions were shut down. This situation created at least a 2-year session gap in my study life and my career got hampered.”*

Participant 6 (a 23-year-old university going student), also explained his feelings this way:

*“My depression started mounting when the pandemic situation got worse. I was so depressed while the nationwide lockdown has been imposed because I lost all my private tuition opportunities. These private tuitions were my only source of income. Besides, my family also faced an economic crisis. All these sufferings mounted my depression and anxiety level and I found myself as a mentally ill a person.”*

Academic pressure.

Apart from relationship break up and pandemic (covid-19) situation, four of our study patients expressed their opinion that academic pressure has also raised anxiety and frustration among them, which in return increased their mental health problems. The increasing academic pressure also changes their medication pattern, treatment, and medicine doses.

Participant 2 (a 22 year old university going student), stated how he got his mental health problem and why it got prolonged:

*“According to the doctors, it was bipolar disorder. Bipolar disorder is also called manic depression as it has two parts- depression and extreme emotional mood swings or mania. The main reason behind my illness was depression and the growing pressure of university admission test examination when I just completed my higher secondary”.*

Besides, Participant 5 (a 24 year old university going student), reported how study pressure could increase mental health problems in a student’s life:

*“During my early days when my mental health-related problems started to grow, I was very much tensed due to my study. I was a meritorious student, and I was always serious about my study and academic results. I and my family had expectations from myself. Concurring with these expectations, during my SSC examination I went under enormous study pressure and my mental illness started to grow”.*

Participant 3 (a 24 year old married informant) stated a slightly different reason behind her mental health problem:

*“In my case along with the academic pressure, tension and overdose of medicine took my mental health problems to a serious level.”*

### The reason behind mental health issues (perspective of caregivers)

Among our 13 interview participants, five were the caregivers of the mental health patients. Among the caregivers, three of them admitted that the main reason behind their closest one’s mental health problem emerged due to their academic pressure, schooling type, mismatch of expectation and academic achievement, and failed romantic relationships.

A caregiver father, Participant 12 (48 years, businessman) asserted how his daughter got mental health problems:

*“We came to know that my daughter’s mental health problem emerged due to her academic pressure. She consulted with her mother about the mental stress she is going through due to her academic activities.”*

Similarly, another caregiver mother, Participant 10 (48 years, housewife) confessed how schooling dissatisfaction may cause mental health problem in a person:

*“Since my son’s early childhood, we wanted him to go to Madrasa, a religious education system, and become a Hafez or Mawlana. Therefore, we admitted him to the nearest Madrasa. My son was not satisfied with his Madrasa education. After some days he returned from there, but we forced him to attend again. He told us many times that he did not like to study there but we did not pay attention to his interest. We forced him to fulfill our expectations. I think our expectations and pressure regarding the expectation gave him stress to comply with his mental health issues.”*

Participant 13 (40 years, housewife), another caregiver also stated how the mismatch between expectations and reality caused mental health-related problems:

*“My son dreamt to study in a Cadet College. He took enormous and hectic pressure to prepare for the admission tests. However, he failed to secure a place in the admission test. Next, he worked hard to get a scholarship for the JSC examination, but he failed to secure a scholarship at this stage. He was stressed with the two failures. At the time of his half-yearly examination in ninth grade, he suffered from high fever and was unable to attend the final exam. These failures in three different exams put him in a great depression. Since then, we observed his growing mental health-related issues onward.”*

Apart from these reasons behind mental health problems, a caregiver, Participant 9 (43 years, housewife) admitted that:

*“My son had a relationship with a girl and suddenly she stopped contacting him which put my son into stress, tension, and frustration. As a result, he started to grow mental health problems within himself.”*

Furthermore, Participant 11 (32 years, businessman), another caregiver, explained that:

*“My daughter suffers from typhoid and got several medications and vaccines because of that. But the vaccination and its side effects increased her mental health issues. Later we came to know that the overdose of medicine caused her mental health-related problems. Her psychiatrist also told us that her growing anxiety caused her to carry serious mental health problems onward.”*

### Patient’s exposure to mental health issues

The qualitative data showed that among the informants who faced mental health problems, all of them showed negative exposure as a mental health problem is considered a serious illness for anyone. The response from the mental health patients and caregivers regarding exposure to mental health issues could be explained under two themes,

1. Depression and loneliness
2. Suicidal tendency

Depression and loneliness.

Generally, the mental health patients face day long anxiety and depression all day. Participant 7 (25 years, university student) explained his feelings this way:

*“When I realized my mental health problem, I was not feeling better. Generally, I started avoiding my regular habits and the work that I loved to do. Even if I forced myself to work, I did not get that motivation and stimulation… I kept myself isolated in darkness. I felt like I was comfortable being isolated. My food habit and body clock were also disrupted. I could understand my changes, my anxiety, and my depression but I was helpless. I also noticed I started losing my temper.”*

Participant 4 (24 years, university student) shared her feelings this way:

*I started feeling sleep related problems as my sleeping time was decreasing gradually. I almost found myself sleepless round the 24 hours. Suddenly, I also found myself getting seriously short-tempered and started yelling and shouting at people for minor issues. I felt lonely and wept most of the time for my situation.*

A caregiver, Participant 9 (43 years, housewife) shared how her son was exposed to mental health-related problems:

*“For approximately 6 months, my son appeared depressed and frustrated. He stopped talking with others, did not take his meal properly. He isolated himself from us and the community.”*

Another caregiver, Participant 10 (48 years, housewife) revealed how her son behaved due to mental health problems:

*“My son confined himself in his room. He stopped talking with others, disliked gatherings, stopped contacting friends, laid on his bed the whole day, and didn’t take baths and meals properly.”*

Suicidal tendency.

Excessive anxiety, frustration, and depression as well as a negative outcome of mental health illness influenced patients’ mental condition which raised their suicidal tendency as well. Participant 1 (22 years, university student) shared his feelings this way:

*“When I realized the presence of my mental health issue, I found myself totally useless, unworthy, and meaningless to breathe in this world. I blamed myself for my misery and found no reason to live. So, I thought about quitting my life”.*

Similarly, Participant 3 (24 years, married informant) also stated that:

*“When my mental illness started, I started feeling irritated, physically and mentally unstable. I wanted to finish all the misery and kill myself for good.”*

A caregiver Participant 11 (32 years, businessman) expressed how his wife became suicidal:

*“My wife was out of her control and started talking abnormally. Several times she told me that, she was not interested to stay with me. Also, she accused herself of her misery and would like to attempt suicide.”*

## Discussion

This study was aimed to explore the underlying causes of mental disorders among the young adults and found three major causes: the breakup of romantic affairs; the emergence of the COVID-19 pandemic and its effects, and academic pressure. The predictors of mental health issues are heterogeneous by nature while the findings also suggest distinctive stories.

### Breakdown of relationship

This study found that two of the mental health patients reported mental illnesses, such as depression and frustration, after getting separated from their romantic partners while attending universities. Gustavson, Knudsen [28] argue that young adults have a higher probability of mental disorders than older adults, largely because of emotional instability. Due to the prevailing nature of education, a co-education system, it allows young men and women to get closer to each other. Sometimes, this intimacy leads to romantic affairs, a common phenomenon. Moreover, this emotional attachment between young adults often creates emotional dependency such as sharing love, guidance, and collaboration. At the university level in Bangladesh, the students get more freedom than they enjoy at the schools and colleges; therefore, they start to think of themselves as adults. In many instances, the breakup causes growing loneliness, and such mental state leads to frustration which ultimately results in depression. In this study, it is documented that some of the participants experienced deep depression and loneliness and such mental state, as they opined, was caused by a breakup with their loved ones. This can easily be understood that emotional disengagement between partners may have deteriorated their mental health conditions. Similar findings are also observed in Mamun, Hossain [29] where it is argued that anxiety and stress were resulting from being in a relationship. Low, Dugas (13) also found similar findings. Likewise, the caregivers of the participants also asserted similar stories regarding mental health issues of the sufferers. The caregivers argued that the breakup of a romantic relationship was responsible for mental health issues such as suffering from deep depression, anxiety, and frustration among the patients. Busuito, Huth-Bocks (15) have found similar findings where attachment in a romantic relationship is found linked with stress disorder. Evidence also suggests that for girls, romantic relationships at early puberty were also responsible for disruptive behavior and psychiatric disorder [14,30]. In contrast, in the context of Singapore, a study observed that parenting typology shapes the quality of the romantic relationship such as commitments, and thus, compromise their adult children [31]. In this regard, social support can be a positive factor for individuals who are suffering from mental health issues [24,32,33].

### COVID-19 and lockdown situation

The findings of the study suggests that mental health problems may also have been triggered because of the outbreak of the COVID-19 pandemic. Researchers have found similar findings and many researchers argued that the pandemic has steered serious mental problems among individuals, irrespective of age, sex, or residence [2–5,7,8]. A higher level of association is observed between the COVID-19 pandemic-induced worries and the mental well-being of individuals [19,20,34]. In this study, it is evident that the participants were suffering from mental health problems due to the prolonged lockdown during the early phase of the COVID-19 pandemic in Bangladesh. Following the first reported case of COVID-19 on 8 March 2020 [35], the government of Bangladesh implemented strict non-therapeutic measures, including ‘social distancing,’ ‘work from home,’ and declared a countrywide lockdown in the disguise of ‘general holidays’ from 26 March 2020 to minimize ‘human-to-human’ transmission and to reduce death tolls [35]. During the prolonged home confinement, most of the activities–whether formal, informal, or non-formal–were motionless, and educational institutions were not exceptions. The ‘new normal’ situation and detachment from friends and families, together with misinformation in the social and electronic media [8] gave rise to unprecedented circumstances not witnessed in Bangladesh over the centuries. During the lockdown, people in Bangladesh, particularly the lower class and the marginal people started losing their financial securities resulting in increased indebtedness [7,9,36]; therefore, they experienced heightened levels of anxiety, and depression [3,6,7] as the people started losing their assets [9,36]. In this study, findings also indicated that the participants have gone through depression because of the uncertainty over their academic and professional life. In addition, the loss of financial independence also reduced the confidence of the participants and led them to depression. A similar situation is also noted in a previous study on university students in Bangladesh [6]. Besides, the absence of face-to-face interaction also led to a disturbance in mental state. During the pre-COVID period, students usually met their friends and could share their ideas, thoughts, joys, and sorrows which might have been helpful to reduce depression, anxiety, and stress. Due to the lockdown, the scope of interpersonal interaction was entirely lost for a longer period, which may be responsible for the heightened incidence and severity of mental health issues as evident in this study. Among the Bangladeshi students, COVID-19 symptoms were also found responsible for deteriorating mental health conditions, which is more severe among younger students than their senior ones [37,38].

### Academic pressure

Apart from romantic failure and the trauma of COVID-19, some other issues that may have disturbed the mental state of the young adults in Bangladesh. Studies suggested that apart from financial deprivation, poor standard of living, and lack of recreational facilities, academic pressure might be responsible for poor mental health [6,39,40]. Landstedt, Bortes (39) identified a clear connection between academic achievement and mental health condition from a gendered perspective. However, DeRoma, Leach [41] opposes the connection between academic performance and depression. In the context of Bangladesh, it is well documented that first-year university students experience higher anxiety and depression due to excessive use of the internet, poor sleep quality and so on [42]. In this study, the participants also shared their mental health issues where one of the root causes is identified as academic pressure. The academic load reshaped their way of life and influenced their mental state. Genetically, not everyone possesses similar levels mental capacity; thus, some may lose hopes and experience mental trauma. Evidence suggests that in Bangladesh, due to mental health issues, the suicidal tendency among the university students is getting higher [29]. One of the participants revealed that he was suffering from hyper depression during the admission test in university. In Bangladesh, admission in a university is considered as a major determinant of future career; thus, students take this test very seriously. Sometimes, it may create mental stress and lead to severe mental health issues. Besides, getting better grades also lead to unhealthy competition that in turn increases anxiety among students. According to the caregivers’ view, mental health issues arose among the participants because of academic pressure, unfulfilled expectations, gaps in expected and actual academic achievements. In Bangladesh, the education system is embedded with personal and social expectations which may be favorable for growing mental disorders. In this regard, reducing parental and social expectations toward students, together more relaxed curriculum development with more innovative and participatory method of education may be helpful for reducing academic pressure. Traditionally, parents in Bangladesh impose their own ambition on their children, neglecting the desire of children, and such situation may raise dissatisfaction and frustration among young adult in life. Furthermore, this study found that unachieved dreams along with parental and social expectations are found liable for increased depression among students. It is argued that reduced academic pressure such as shorter class lectures, more group activities and innovative approaches can be helpful to reduce depression, anxiety, and stress among the students [40]. In addition, supportive activities and interventions are also recommended for reducing mental health problems among students in Bangladesh.

## Limitations

The selection process of the study location and informants does not provide a full representation, as the study location and informants represent a small proportion of a larger population. Therefore, the findings cannot be generalized for a wider group of mental health patients in Bangladesh. The selection bias of students receiving mental healthcare and caregivers may also limit the generalizability. Other restrictions we must address include problems with funding, a lack of time, and communication difficulties during the lockdown of COVID-19. We also acknowledge that further data from a wider range of patients from around the nation would be necessary to declare the findings as generally applicable. Yet, this study would potentially contribute to mental health literature further in the context of Bangladesh.

## Conclusions

This study aimed to explore the underlying causes of growing mental health problems among young adults, and the findings suggested that failure in romantic relationship, academic pressure, and pandemic-induced circumstances adversely affected the mental wellbeing of young adults in Bangladesh. Therefore, it is strongly recommended that government and its policymakers devise age-specific and individualized mental health services (e.g., student counselling through psychotherapy, pharmacotherapy and alternative care strategies) as well as psychoeducational materials through psychiatrists and other mental health practitioners in educational institutions, including schools, colleges, and universities, in order to timely address the mental health issues among young adults to make sure uninterrupted educational progress that would pave ways for successful professional careers [19,20,22,43,44]. Moreover, social acceptance of mental health-related problems, such as a positive approach in dealing with mental health problems from the families or communities is also required [24]. Academics should direct more empirical research on academic environment and mental wellbeing of students to ensure quality and inclusive education without imposing burden.

## Supporting information

S1 Appendix (DOCX)
