# Supplementary material for: A qualitative investigation of stressful life events and mental health disorders: The views of patients and caregivers in Bangladesh
Source: PLoS One. 2023 Feb 9;18(2):e0281649. doi: 10.1371/journal.pone.0281649 (PMC9910653; doi:10.1371/journal.pone.0281649)
Supplement: S1 Appendix — (DOCX) [file pone.0281649.s001.docx]

# Appendix

**The social stigma against mental health issue: An exploratory investigation in a southwestern city of Bangladesh**

**Section One: Socio-demographic information**

1.1 Name of Participants: 1.2 Age:

1.3 Sex: 1.4 Religion:

1.5 Education: 1.6 Marital Status:

1.7 Living arrangement: 1.7 Occupation:

1.8 Income: 1.9 Location of residence:

1.10 District:

**Section Two: Household Information**

2.1 Family type: 2.2 Family composition:

2.3 Household head: 2.4 Education of HHH:

2.5 Occupation of HHH: 2.6 Income of HHH:

2.7 Income of HH: 2.8 Sources of Income of HH:

2.9 Assets or SES of HH: 2.10 Earning members of HH:

2.11 Expenditure of HH

**Section Three: Mental health experience and social response – the patient’s perspective**

3.1 When did you realize the presence of a mental health issue? What was it? When did it start? What could possibly trigger the mental health problem? Did your family members ever experience such mental health issues?

3.2 How long have you been going through the problems? What was your immediate response? Did you seek counselling from psychiatrist immediately, or you opted to share your problems with family or friends? What was the response from your family members regarding the mental ailment? Did your family bare you to share the problems with others or did they accept the issue without any prejudice? Did you face any sort of labelling or are you being stigmatized by your family, friends or community members?

3.3 How long did it take to you to seek mental health counselling from psychiatrist? Did you consult with the psychiatrist your own, or your family members took you for the psychological session? What was the response of your psychiatrist? Are you going through medication or counselling only or both? Do you think that you are improving or is it going to worse? Are you satisfied with the support from the family and the treatment from the psychiatrist?

3.4 Based on your experience, tell us what you think about the mental health problems, what are the major causes of mental health issues and what could be the remedies of mental health problems in Bangladesh? Do you think that support from family and community is necessary to address to minimize mental health-related stigma?

**Section Four: Mental health experience and social response – the caregiver’s view**

4.1 What is your relationship with the patient? How long have you been known the patient?

4.2 What is the mental problem of the patient and what are the symptoms? When did the mental health issue emerged? What could be the reason behind the mental health problem? How long did the patient suffer from the mental ailment?

4.3 Did the family members realize the presence of mental health issue or did the patient explain it to the family members? Do you know any history of mental ailment among other family members in the past?

4.4 What was the immediate response from the family members? What measures did they take to deal with the mental health issue of the patient? Did they bare the patient to interact with others in your family or your community? How did your community react and how did your family manage the social reaction?

4.5 When did you seek assistance from the psychiatrist? Did you or family members bring the patient here or others compelled you to do so? Do you think the patient is improving and the ailment is getting worse? Are you satisfied with the treatment?

4.6 What are the major challenges did you family face to deal with a mental patient in your family? How did your family deal with the backlash (if any) from the community? Did the patient or other family members being labelled or stigmatized by the community? Did you get any support from the community?

4.7 Do you think the mental health problems are getting sufficient attention? What do you suggest minimizing the mental problems in Bangladesh, in general?
